# P-1552. Risk Factors For Multidrug-Resistant *Klebsiella Pneumonia* Urinary Tract Infections

**DOI:** 10.1093/ofid/ofae631.1719

**Published:** 2025-01-29

**Authors:** Fatma Hammami, Khaoula Rekik, Fatma Smaoui, Amal Chakroun, Chakib Marrakchi, Makram Koubaa, Mounir Ben Jemaa

**Affiliations:** Infectious Diseases Department, Hedi Chaker University Hospital, University of Sfax, Tunisia, Sfax, Sfax, Tunisia; Infectious Diseases Department, Hedi Chaker University Hospital, University of Sfax, Tunisia, Sfax, Sfax, Tunisia; Infectious Diseases Department, Hedi Chaker University Hospital, University of Sfax, Tunisia, Sfax, Sfax, Tunisia; Infectious Diseases Department, Hedi Chaker University Hospital, University of Sfax, Tunisia, Sfax, Sfax, Tunisia; Infectious Diseases Department, Hedi Chaker University Hospital, University of Sfax, Tunisia, Sfax, Sfax, Tunisia; Infectious Diseases Department, Hedi Chaker University Hospital, University of Sfax, Tunisia, Sfax, Sfax, Tunisia; Infectious Diseases Department, Hedi Chaker University Hospital, University of Sfax, Tunisia, Sfax, Sfax, Tunisia

## Abstract

**Background:**

Multidrug-resistant (MDR) *Klebsiella pneumonia* urinary tract infections (UTI) has increased over the past years, leading to problems in treatment and management. We aimed to study the risk factors associated with MDR *Klebsiella pneumonia* UTI.

**Methods:**

We conducted a retrospective study including all patients hospitalized in the infectious diseases department for UTI caused by *Klebsiella pneumonia* between 2015 and 2022.

**Results:**

We encountered 134 cases among whom 78 cases were MDR (58.2%) and 56 cases were non-MDR (41.8%). Patients with MDR UTI were significantly older (62±19 years vs 47±21 years; p< 0.001). Patients aged 60 years and above (70.5% vs 37.5%; p< 0.001) and male patients (46.2% vs 26.8%; p=0.023) were significantly more affected by MDR UTI. Diabetes mellitus (41% vs 21.4%; p=0.017) and renal calculi (19.2% vs 5.4%; p=0.02) were significantly more frequent among MDR UTI cases. Recent invasive urologic procedure (21.8% vs 3.6%; p=0.003), previous medical history of MDR UTI (24.4% vs 7.1%; p=0.009), antibiotics use (53.8% vs 17.9%; p< 0.001) and previous hospitalization (42.3% vs 12.5%; p< 0.001) were significantly more frequent among MDR UTI cases. C-reactive protein levels were significantly lower among MDR UTI cases (40[5-155] mg/l vs 224[143-288] mg/l; p=0.002). A favorable evolution of the disease was significantly more frequent among non-MDR UTI cases (87.5% vs 67.9%; p=0.009). As to complications, death and relapse, no significant difference was noted.

**Conclusion:**

Male diabetic patients at an advanced age, with previous medical history of MDR UTI, renal calculi or recent invasive urologic procedure were at high risk of acquiring MDR UTI caused by *Klebsiella pneumonia.*

**Disclosures:**

**All Authors**: No reported disclosures

